# Supplementary material for: Cultivation of marine bacteria of the SAR202 clade
Source: Nat Commun. 2023 Aug 22;14:5098. doi: 10.1038/s41467-023-40726-8 (PMC10444878; doi:10.1038/s41467-023-40726-8)
Supplement: Supplementary file 1 — Supplementary Information [file 41467_2023_40726_MOESM1_ESM.pdf]

Supplementary Information for

Cultivation of Marine Bacteria of the SAR202 Clade

Yeonjung Lim[1,2], Ji-Hui Seo[1], Stephen J. Giovannoni[3], Ilnam Kang[2*], and Jang-Cheon Cho[1*]

[1]Department of Biological Sciences and Bioengineering, Inha University, Incheon 22212, Republic of Korea

[2]Center for Molecular and Cell Biology, Inha University, Incheon 22212, Republic of Korea

[3]Department of Microbiology, Oregon State University, Corvallis, OR 97331, USA

*Correspondence:

Ilnam Kang (ikang@inha.ac.kr) and Jang-Cheon Cho (chojc@inha.ac.kr)

Supplementary Notes 1

Genome-based phylogeny of the SAR202 clade 1

Genomic inference of the metabolic potentials 2

Supplementary Figures 1 To 9 4

Supplementary Tables 1 To 4 13

Supplementary References 18

Supplementary Notes

Genome-based phylogeny of the SAR202 clade

Genome-based phylogeny showed that the entire SAR202 clade corresponds to a monophyletic superorder in the Genome Taxonomy Database (GTDB), with the SAR202 strains of this study belonging to the SAR202 group I, which corresponds to an order in the GTDB. To find the phylogenomic position of the four SAR202 strains in the context of both the previously proposed SAR202 groups (I to VII) and the GTDB[1-3], we classified SAR202 genomes (the four genomes of this study and other SAR202 MAG/SAGs reported previously) using GTDB-Tk, and then constructed a phylogenomic tree that encompasses GTDB taxa into which the SAR202 genomes were classified. The resulting tree (Fig. 1b) confirmed the phylogenetic position of the four strains analyzed by 16S rRNA gene phylogeny: the four strains belonged to the SAR202 group I, which corresponds to o__UBA1151 in the GTDB taxonomy (more specifically, group Ia corresponding to f__Bin127 in the GTDB R202). The whole SAR202 clade was revealed to be a monophyletic superorder (~10 orders) of the class *Dehalococcoidia* in the GTDB taxonomy, with most SAR202 groups corresponding to one of the orders, except for group V containing two orders. The order named "o__SAR202" in the GTDB is just one of multiple orders comprising the whole SAR202 clade. The orders SAR202-VII-2, GCA-2717565, and UBA1127 do not correspond to the groups of the SAR202 clade, but they are regarded to belong to the SAR202 clade, because these orders were nested within the orders corresponding to the SAR202 groups. The description of the SAR202 clade as a superorder is also consistent with a previous analysis, which showed a within-clade 16S rRNA gene sequence similarity as low as 78.7%[1], which is slightly higher than the threshold for class as proposed by Yarza *et al*[4]. This enormous phylogenetic diversity of the SAR202 clade may underlie metabolic and ecological divergence among the SAR202 groups reported previously[2].

Genomic inference of the metabolic potentials

The JH545 genome exhibits features of a heterotrophic lifestyle (Fig. 3), in accordance with previous studies on SAR202 MAG/SAGs. The genome has genes for central carbon and energy metabolism of typical aerobic organoheterotrophs, including the Embden-Meyerhof-Parnas glycolytic pathway, the tricarboxylic acid (TCA) cycle, oxidative and non-oxidative pentose phosphate pathway (PPP), and electron transport chain (except for cytochrome *c* reductase). Gluconeogenesis is incomplete due to the lack of fructose-1,6-bisphosphatase. Several features found in central metabolic pathways are as follows. In accordance with a previous study on SAR202 MAGs[5], decarboxylation of pyruvate to acetyl-CoA is mediated by both pyruvate dehydrogenase complex and pyruvate:ferredoxin oxidoreductase. In contrast to pyruvate, 2-oxoglutarate, a key intermediate of TCA cycle, is decarboxylated by only 2-oxoacid:ferredoxin oxidoreductases. Alpha-ketoglutarate (2-oxoglutarate) dehydrogenase complex was not predicted in the genomes. The first step of oxidative PPP is mediated by glucose-6-phosphate dehydrogenase (NAD^+) (EC 1.1.1.388), instead of more generally used glucose-6-phosphate dehydrogenase ($NADP^+$) (EC1.1.1.49). The enzymes assigned to EC 1.1.1.388 belong to a novel type of glucose-6-phosphate dehydrogenase discovered in a recent study that demonstrated oxidative PPP for the first time in the domain Archaea[6]. This novel enzyme group, assigned to K19243 in the KEGG database, is not related to the enzymes belonging to EC 1.1.1.49 (K00036). A search for K19243 proteins in representative genomes for species clusters of the GTDB (R95), conducted using AnnoTree, revealed that 3.8% (1,135 of 30,238) and 18.2% (304 of 1,672) of bacterial and archaeal genomes possessed this novel enzyme, respectively. While rare in bacteria, this novel enzyme is found in all genomes of o__UBA1151, suggesting the importance of this enzyme in carbon metabolism of SAR202 subgroup I.

Like many marine heterotrophs[7], the SAR202 genomes were predicted to be deficient in the biosynthetic pathways of several vitamins, including thiamine (B_1), biotin (B_7), and cobalamin (B_{12}). The lack of thiamine phosphate synthase, in addition to the biosynthetic pathways of the precursors, suggested a requirement for intact B_1. Only a salvage pathway was found for cobalamin. Folate (B_9) biosynthesis is also questionable due to the absence of several steps including those for biosynthesis of 4-aminobenzoate, a key precursor of folate. Also, it was predicted that the biosynthetic pathways for serine and cysteine could be incomplete due to the lack of *serB* (phosphoserine aminotransferase) or *cysE* (serine O-acetyltransferase), which would require wet experiments for verification.

In addition to genes functioning in aerobic heterotrophic metabolism, some genes involved in lithotrophy and anaerobic respiration were also found in the JH545 genome. Strain JH545 is predicted to use sulfide as electron donor. Sulfide:quinone oxidoreductase (EC 1.8.5.4), which directly transfers electrons from sulfide to the quinone pool, was predicted to be present in the genome, suggesting the possibility of utilizing sulfide as an energy source[8]. In contrast to previous studies[5,9], genes necessary for energy conservation by oxidation of sulfite or methanesulfonate were not found. Although lithoautotrophy is unlikely due to the absence of any carbon fixation pathways, the energy from sulfide would contribute to survival in oligotrophic habitats. In terms of electron acceptors, nitrate reductase (NapAB) and N2O reductase (NosZ) were predicted. Although no energy conservation is possible since both enzymes are periplasmic, utilization of these nitrogen compounds as electron acceptors may provide an advantage in anaerobic conditions that may be encountered in some habitats. The biosynthetic pathway for molybdenum cofactor (Moco), an essential coenzyme of nitrate reductase, and the ABC transporter for molybdate were also predicted to be encoded in the genome.

Supplementary Figures

a

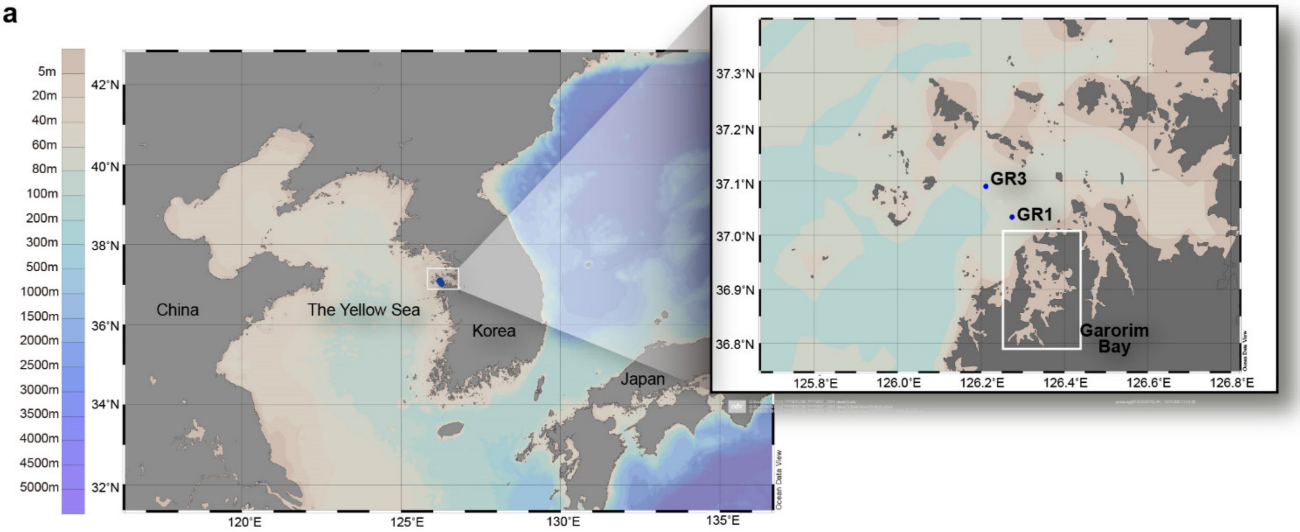

b

Station	Date	Latitude/ Longitude	Temperature (℃)	Conductivity (mS cm^{-1})	Salinity (‰)	DO (%)	DO (mg L^{-1})	ORP (mV)	PO_4^{3-} (mg L^{-1})	SiO_2 (mg L^{-1})	NH_4^+ (mg L^{-1})	NO_2^- (mg L^{-1})	NO_3^- (mg L^{-1})
**GR1**	2017.10.16	37°02'00.6"N 126°16'29.7"E	19.6	48.3	31.6	77.0	5.9	306.0	0.0237	0.4008	0.0081	0.0464	0.0361
**GR3**	2017.10.16	37°05'23.9"N 126°12'43.4"E	19.4	48.8	31.9	76.1	5.8	314.4	0.0206	0.3419	0.0198	0.0400	0.0282

Supplementary Fig. 1 | Map of the sampling stations and physicochemical parameters of the seawater samples used for HTC and amplicon sequencing. a, Map of the sampling stations. The two blue dots indicate the sampling stations, GR1 and GR3, located outside of the Garorim Bay in the Yellow Sea. The map was created by Ocean Data View program[10]. **b,** Physicochemical parameters of the seawater samples used for HTC and amplicon sequencing. Temperature, conductivity, salinity, dissolved oxygen (DO), and oxidation reduction potential (ORP) were measured using a portable instrument (YSI Model 556, YSI Incorporated). Concentrations of inorganic nutrients were determined using a QuAAtro microflow analyzer (SEAL Analytical).

a

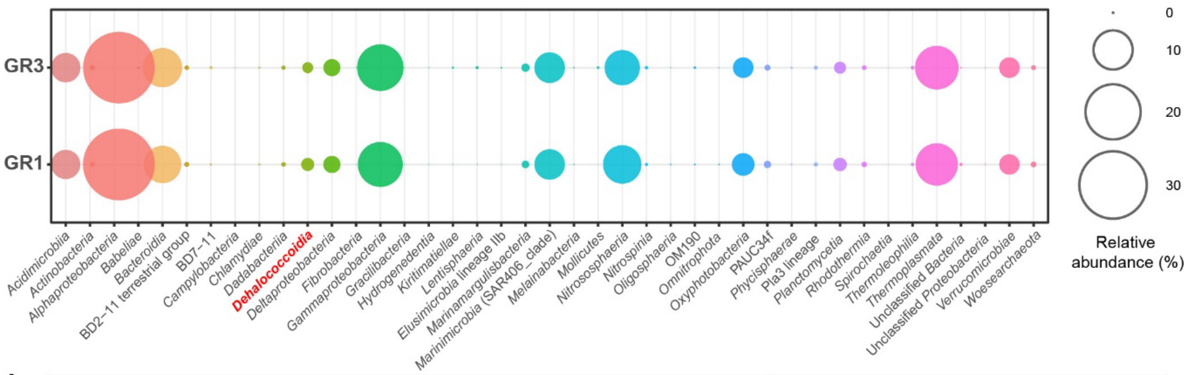

b

SAR202 ASVs (feature name by Qiime2)	Number of reads		Similarity to JH545	Putative GTDB taxonomy assigned by BlastN
	GR1	GR3	(%)	against SSU rRNA gene sequneces of GTDB (R202)
77457246f9062ccd04d4e36179dbb330	321	245	100.0	o__UBA1151;f__Bin127;g__UBA1328;s__UBA1328
7b6f78add5027e069ea749ba1125ece6	107	101	87.2	o__UBA1151;f__TMED-70;g__TMED-70;s__TMED-70
50071d6774704b2c5470ad88729a212c	27	6	96.5	o__UBA1151;f__Bin127;g__UBA9455;s__UBA9455
93848f4d2f0a73caccae0e4658690eca	8	8	84.3	o__UBA1151;f__TMED-70;g__GCA-002700125;s__GCA-002700125
Number of SAR202 reads	463	360		
Number of total reads	44144	49751		

Supplementary Fig. 2 | Prokaryotic community composition of the seawater samples used for the HTC experiment, collected at sampling stations GR1 and GR3. The sequencing data of 16S rRNA gene amplicon were analyzed by DADA2 plugin of QIIME2. Taxonomic classification of ASVs was based on SILVA SSU database (release 138). **a,** Relative abundance of prokaryotic groups at class level. Most of the reads classified into *Dehalococcoidia* (indicated in red) belonged to the SAR202 clade. Source data are provided as a Source Data file. **b,** The number of sequencing reads assigned to the four SAR202 ASVs in each seawater sample. For each ASV, sequence similarities to JH545 and the putative GTDB taxonomy assigned based on BlastN against the SSU rRNA gene sequences of GTDB (R202) are also indicated. All the four ASVs were assigned to an order (d__Bacteria; p__Chloroflexota; c__Dehalococcoidia; o__UBA1151) corresponding to the SAR202 group I.

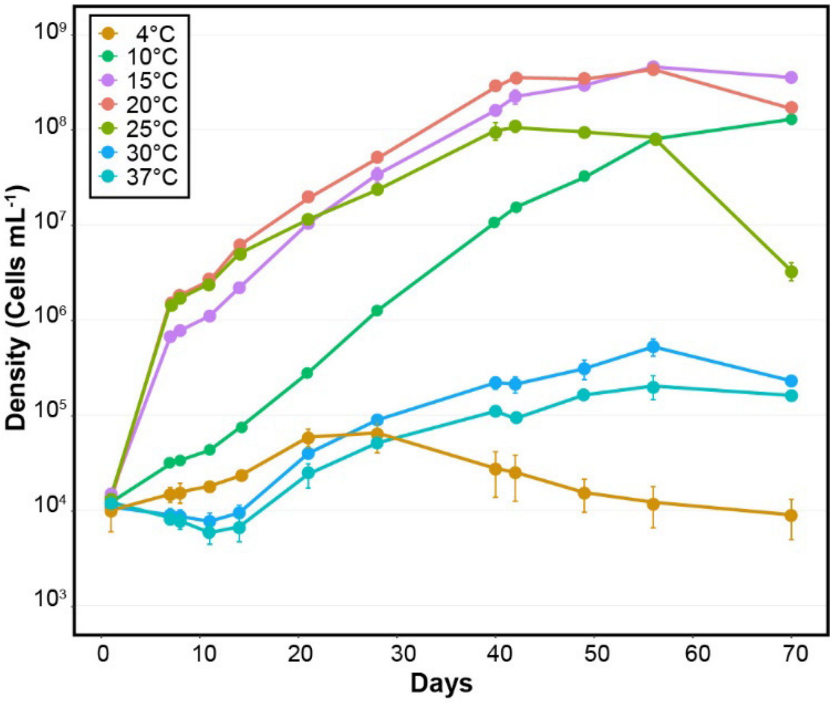

Supplementary Fig. 3 | Growth curves of strain JH545 at various temperatures. Cultures were incubated in LNHM in dark at temperatures ranging between 4 °C and 37 °C. Experiments were performed in duplicates. Data are presented as mean values ± SEM. Note that the error bars are hidden when they are shorter than the size of the symbols. Source data are provided as a Source Data file.

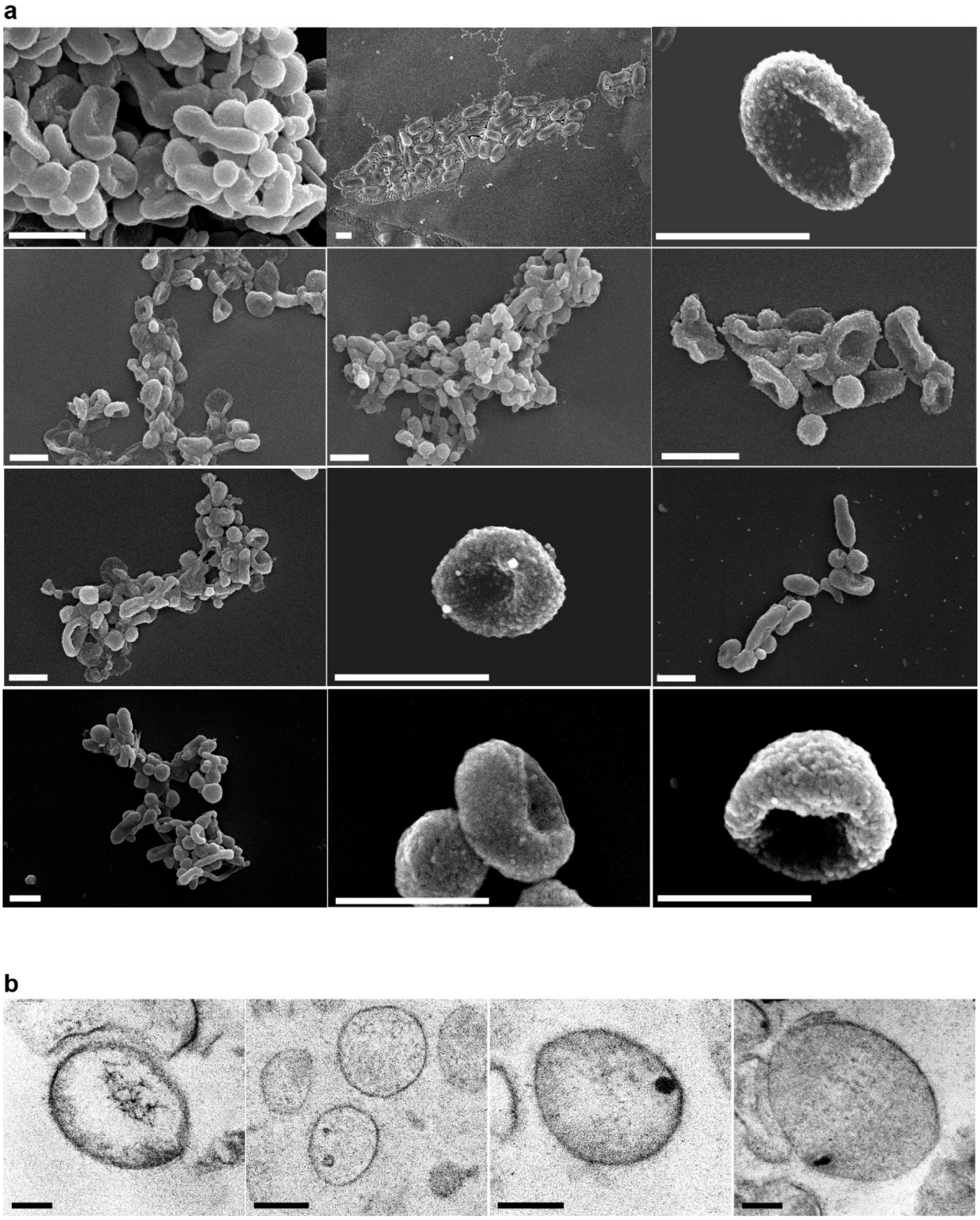

Supplementary Fig. 4 | Morphology of strain JH545 cells observed by SEM (a) and thin-section TEM (b). Scale bars represent 1 μm and 200 nm in panels (a) and (b), respectively.

a

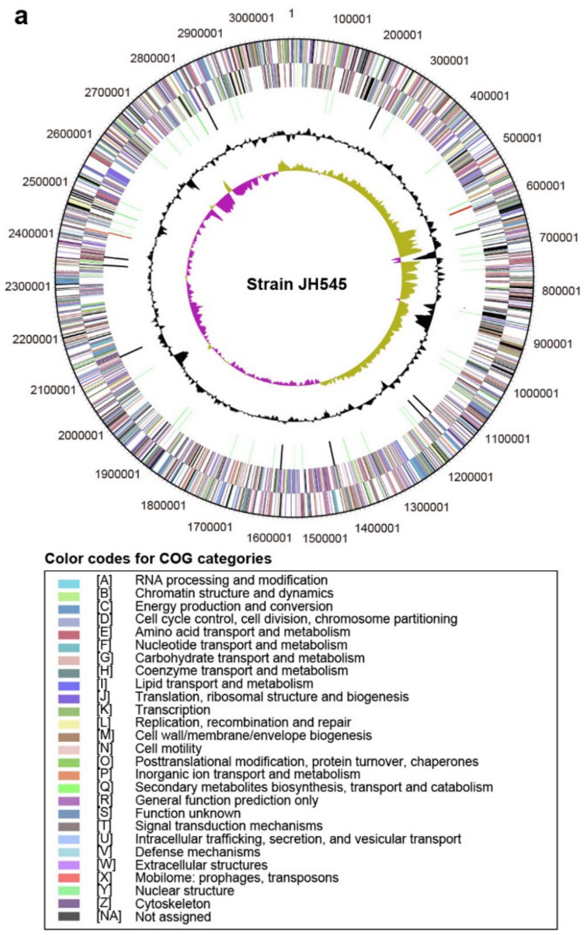

b

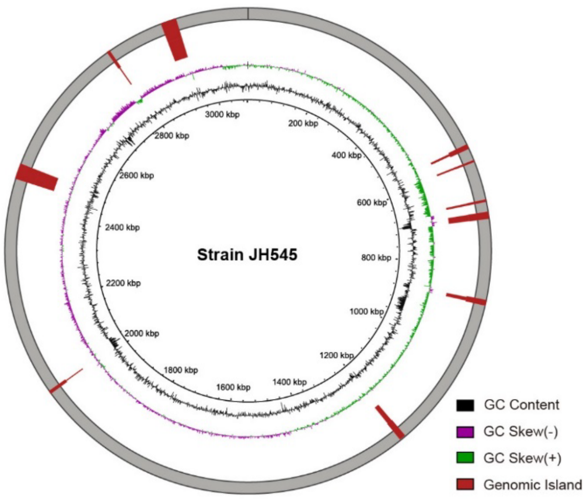

c

	JH545	JH639	JH702	JH1073
**Sequencing**				
Sequencing platform	PacBio RS II (P6-C4 chemistry)	Illumina HiSeq (2 × 150 bp)	Illumina HiSeq (2 × 150 bp)	PacBio RS II (P6-C4 chemistry)
Fold coverage	188.65 ×	339.35 ×	460.57 ×	282.48 ×
**Genome features**				
Size (bp)	3,091,874	3,083,284	3,084,127	3,094,136
Number of contig	1	31	33	1
DNA G + C content (%)	51.81	51.78	51.78	51.78
CDS	2874	2880	2867	2890
rRNA genes	3	3	3	3
tRNA genes	51	51	49	51
CDS assigned to COG	2273	2261	2264	2277
CDS assigned to Pfam	2297	2288	2289	2303
CDS assigned to KO	1212	1197	1198	1215

Supplementary Fig. 5 | Features of the four SAR202 genomes. a, Genome map of strain JH545. From outside to the center: Genes on the forward strand (colored by COG categories), genes on the reverse strand (colored by COG categories), RNA genes (tRNAs in green, rRNAs in red, other RNAs in black), GC content, and GC skew. This genome map was produced by IMG-ER annotation. **b,** Genomic islands of the JH545 genome predicted by IslandViewer 4. The two inner rings showing GC content and GC skew were drawn by BRIG (BLAST ring image generator). The outermost ring shows the positions of genomic islands predicted by several methods implemented in IslandViewer 4. **c,** Genome statistics of the four SAR202 strains. Annotation was performed by the IMG-ER pipeline.

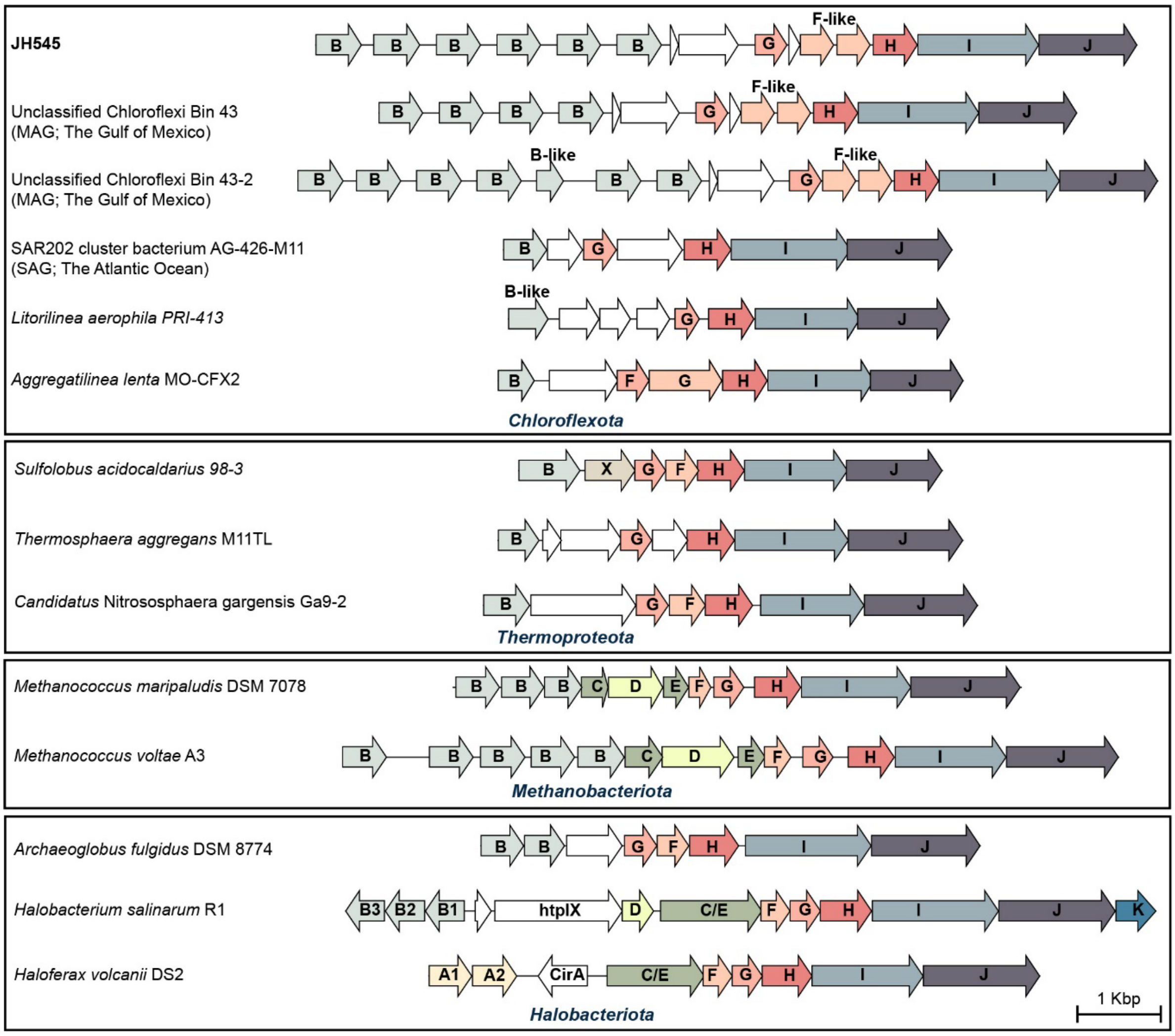

Supplementary Fig. 6 | Map of archaella gene clusters found in the JH545 genome, three SAR202 MAG/SAGs, two *Chloroflexota* isolates, and several archaeal genomes. Gene names are shown inside the arrows representing genes. Names of archaella genes are shown with a single capital letter that corresponds to the letter "X" in the gene name "flaX", *i.e.*, the last letter of the gene name. Homologous genes are shown in the same color. Genes of unknown function are depicted in white. The flaB-like and flaF-like genes of the SAR202 genomes show sequence similarity to the respective archaeal homologs but were not annotated clearly. Genomes are grouped by their phylum-level affiliations according to the GTDB. Easyfig was used to generate the figure.

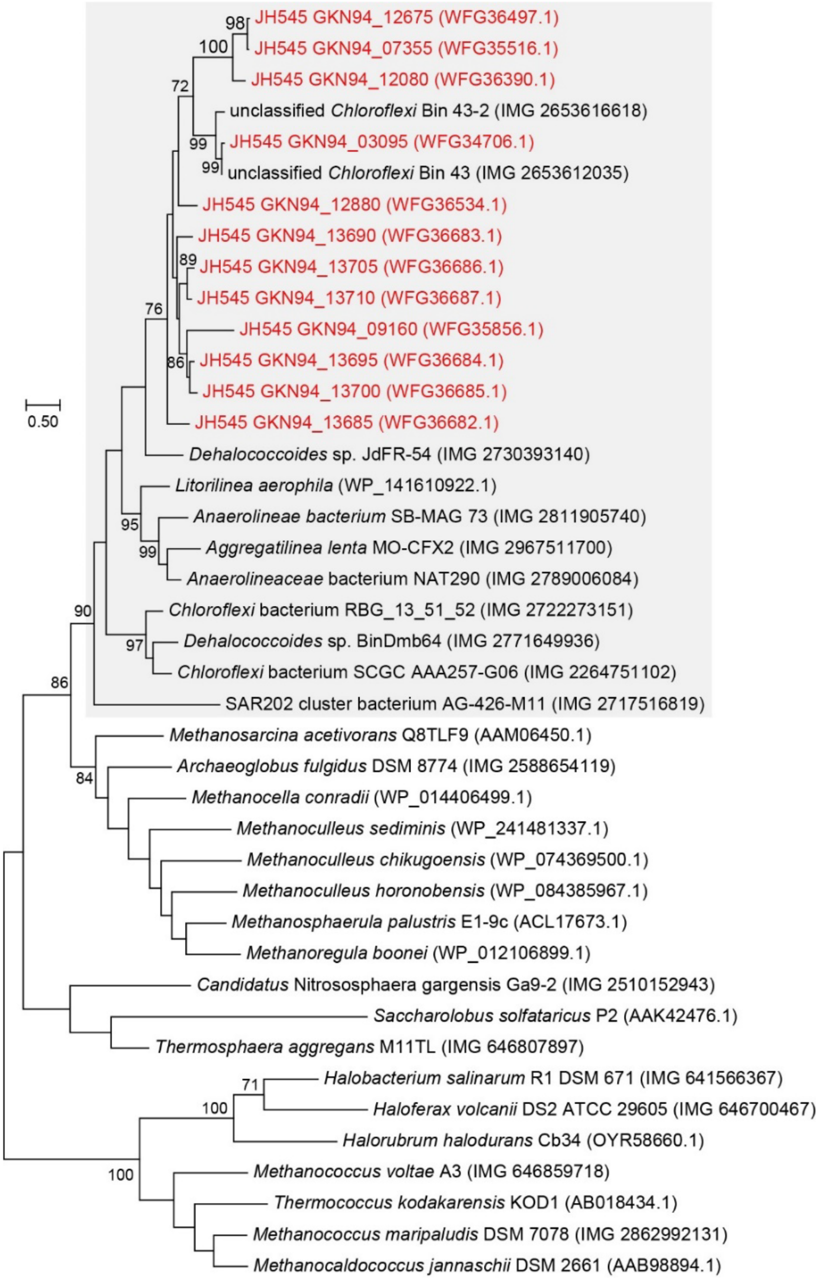

Supplementary Fig. 7 | A Phylogenetic tree of FlaB proteins. A maximum likelihood phylogenetic tree was built using RAxML (v8.2.12) with the PROTGAMMAAUTO option, after sequence alignment by MUSCLE. This tree includes FlaB sequences from strain JH545 (12 sequences; in red), phylum *Chloroflexota* (shaded in gray), and domain *Archaea*. GenBank accession numbers or IMG gene IDs of the sequences are indicated in parentheses following strain names. Except JH545, only one sequences per each genome were included. When multiple FlaBs were retrieved from the same genomes, only one that showed the highest similarity to one of JH545 FlaB (IMG gene ID: 2901385779) was included. The tree was midpoint rooted. Bootstrap support values (≥70%, from 100 resamplings) are shown. Bars, 0.50 substitutions per amino acid position.

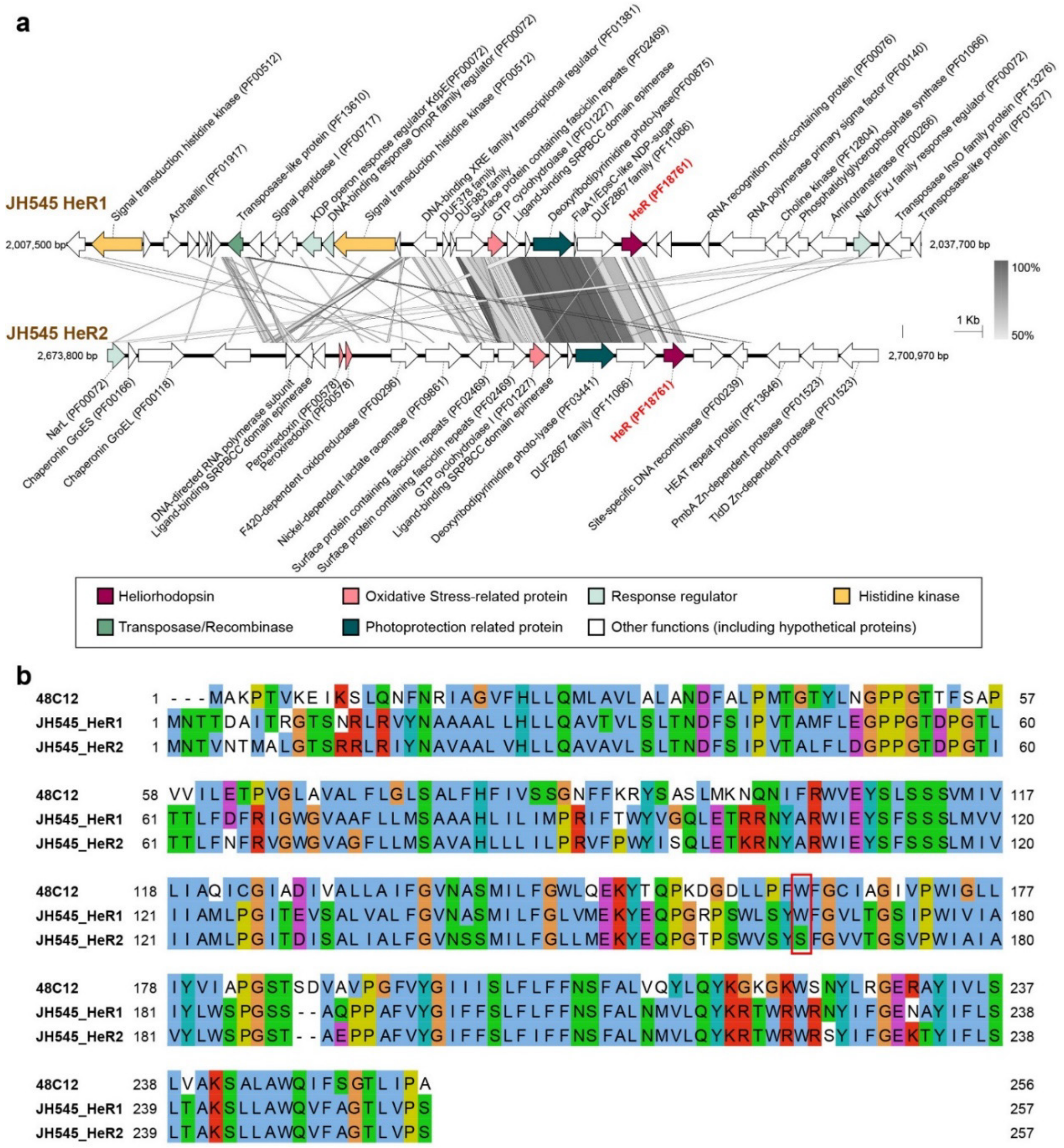

Supplementary Fig. 8 | Heliorhodopsin (HeR) in the JH454 genome. a, Genomic context around the two heliorhodopsin genes of the JH545 genome. Genes putatively assigned to several functions are indicated by colors following the legend at the bottom. Grey boxes between the two genomic regions indicate nucleotide-level similarity following the scale at middle right. This map was drawn using Easyfig. **b,** Amino acid sequence alignment of HeR proteins. The two HeR proteins of JH545 and the first characterized HeR (48C12) were aligned by Clustal Omega and visualized by Jalview with Clustal X color scheme. The red box indicates the amino acid position (W163 in 48C12) where the two HeR of JH545 have different residues and replacement with alanine caused a spectral shift in 48C12.

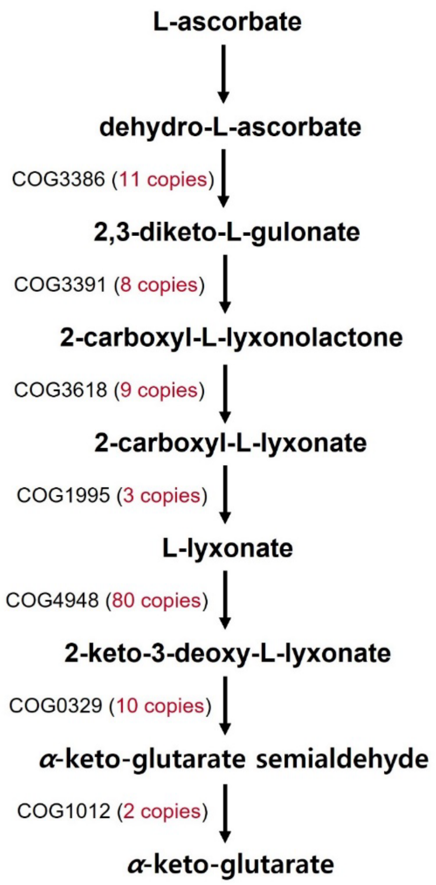

Supplementary Fig. 9 | Catabolic pathways of L-ascorbate inferred from SAR202 genome annotation. This non-phosphorylative pathway is based on a KEGG pathway map (ascorbate and aldarate metabolism; map00053) and MetaCyc pathways (L-ascorbate degradation and L-lyxonate degradation). The number of proteins assigned to the COGs are indicated within parentheses next to the COG IDs.

Supplementary Tables

Supplementary Table 1 | The compositions of low-nutrient heterotrophic medium (LNHM) and artificial seawater medium (ASW) used in this study.

Compound		Final concentration
**Base salts**		
**LNFM**	0.2 um-filtered and autoclaved seawater	
**ASW**		
	NaCl	423.0 mM
	$MgCl_2$	22.9 mM
	$CaCl_2$	9.3 mM
	KCl	9.0 mM
	$MgSO_4$	25.5 mM
	$NaHCO_3$†	2.1 mM
	NH_4NO_3	0.020 mM
	Na_2HPO_4	0.056 mM
**Macronutrients**		
	NH_4Cl	100 µM
	KH_2PO_4	10 µM
**Trace metals**		
	$FeCl_3$	117 nM
	$MnCl_2$	9 nM
	$ZnSO_4$	800 pM
	CoCl2	500 pM
	Na_2MoO_4	300 pM
	Na_2SeO_3	1 nM
	$NiCl_2$	1 nM
**Vitamin mixture**		
	Thiamine (B1)	590 nM
	Niacin (B3)	810 nM
	Pantothenate (B5)	840 nM
	Pyridoxine (B6)	590 nM
	Biotin (B7)	4 nM
	Folic Acid (B9)	5 nM
	Vitamin B12	700 pM
	Inositol	6 µM
	Aminobenzoic Acid	70 nM
**Carbon mixture**		
	D-Glucose	50 µM
	Sodium pyruvate	100 µM
	D-Ribose	50 µM
	N-acetyl-d-glucosamine	50 µM
	Glycerol	50 µM
	Methylamine	50 µM
**Amino acid mixture**		
	20 proteinogenic amino acids	100 nM, each

† $NaHCO_3$ was added after autoclaving to avoid precipitation.

* Macronutrients, trace metals, vitamin mixture, carbon mixture, and amino acid mixture were added after autoclaving.

*In both LNHM5x and ASW5x media, the concentrations of the macronutrients, trace metals, vitamin mixture, carbon mixture, and amino acid mixture were increased by 5-fold.

Supplementary Table 2 | Taxonomic distribution of the 610 isolates obtained by HTC experiments. Taxonomic classification was performed using Mothur (v1.39.5; "classify.seqs" command) based on SILVA SSUREF database (release 138) and is presented at the family level. Sampling stations and culture conditions (catalase addition, dark vs. light) are also indicated. Note that "_or" and "_fa" in some order and family names are suffixes indicating the taxonomic level of the taxa.

				**GR1**				**GR3**				
				No Catalase		Catalase		No Catalase		Catalase		**SUM**
**Phylum**	**Class**	**Order**	**Family**	Dark	Light	Dark	Light	Dark	Light	Dark	Light	
Actinobacteria	*Acidimicrobiia*	*Actinomarinales*	*Actinomarinaceae*	0	0	1	0	0	0	0	0	**1**
		Microtrichales	*Ilumatobacteraceae*	0	0	0	1	0	0	1	0	**2**
	Actinobacteria	*Actinomycetales*	*Actinomycetaceae*	0	0	1	0	0	0	0	0	**1**
		Micrococcales	*Micrococcaceae*	0	0	0	0	1	0	0	0	**1**
		Propionibacteriales	*Propionibacteriaceae*	2	0	0	0	0	0	0	0	**2**
	Thermoleophilia	*Gaiellales*	uncultured	0	2	0	0	0	1	0	2	**5**
Armatimonadetes	*Fimbriimonadia*	*Fimbriimonadales*	*Fimbriimonadaceae*	0	0	0	0	2	2	2	0	**6**
Bacteroidetes	*Bacteroidia*	*Bacteroidales*	*Prevotellaceae*	0	0	0	0	0	1	0	0	**1**
		Chitinophagales	*Chitinophagaceae*	0	0	0	0	1	0	2	0	**3**
			Saprospiraceae	0	0	0	0	0	0	1	0	**1**
		Flavobacteriales	*Crocinitomicaceae*	0	0	0	0	0	0	1	0	**1**
			Flavobacteriaceae	0	0	0	0	0	0	0	1	**1**
			NS9_marine_group	0	0	0	0	0	0	1	0	**1**
Chloroflexi	***Dehalococcoidia***	**SAR202_clade**	**SAR202_clade_fa**	**7**	**0**	**7**	**0**	**7**	**0**	**3**	**0**	**24**
Cyanobacteria	*Melainabacteria*	*Obscuribacterales*	Obscuribacterales_fa	1	0	0	0	0	0	2	0	**3**
	Oxyphotobacteria	*Nostocales*	uncultured	0	0	0	0	0	0	1	0	**1**
Deinococcus-Thermus	*Deinococci*	*Deinococcales*	*Deinococcaceae*	1	0	0	0	1	0	1	0	**3**
Firmicutes	*Bacilli*	*Bacillales*	*Paenibacillaceae*	0	0	0	0	0	0	0	1	**1**
			Staphylococcaceae	0	0	0	0	0	0	0	1	**1**
	Clostridia	*Clostridiales*	Family_XI	0	0	0	0	1	0	0	0	**1**
			Ruminococcaceae	0	0	0	0	1	0	0	0	**1**
Lentisphaerae	*Lentisphaeria*	*Lentisphaerales*	*Lentisphaeraceae*	0	0	0	0	1	0	0	0	**1**
Planctomycetes	OM190	OM190_or	OM190_fa	0	0	1	0	0	0	0	0	**1**
	Phycisphaerae	*Phycisphaerales*	*Phycisphaeraceae*	1	1	0	0	0	0	0	0	**2**
Proteobacteria	*Alphaproteobacteria*	*Caulobacterales*	*Caulobacteraceae*	0	0	0	0	0	1	0	0	**1**
		Puniceispirillales	EF100-94H03	0	0	0	0	1	0	0	0	**1**
			SAR116_clade	1	0	1	0	1	0	0	0	**3**
		Rhizobiales	*Beijerinckiaceae*	0	0	0	0	1	0	1	0	**2**
			Rhizobiaceae	1	0	0	0	0	0	0	0	**1**
			Xanthobacteraceae	0	0	0	0	0	0	1	0	**1**
		Rhodobacterales	*Rhodobacteraceae*	13	6	9	1	11	2	8	1	**51**
		Rhodospirillales	AEGEAN-169_marine_group	8	4	11	2	8	0	5	1	**39**

		SAR11_clade	Clade_I	16	6	17	4	20	9	35	16	**123**
			Clade_III	0	1	0	0	0	0	0	0	**1**
		Sphingomonadales	*Sphingomonadaceae*	0	0	0	0	0	1	3	1	**5**
		uncultured_alphaproteobacteria	uncultured_fa	1	0	3	0	2	0	1	0	**7**
	Oligoflexia	*Oligoflexales*	0319-6G20	0	0	0	0	1	0	1	0	**2**
	Betaproteobacteria	*Burkholderiales*	*Burkholderiaceae*	0	1	2	0	0	1	0	0	**4**
		Methylophilales	*Methylophilaceae*	13	4	7	1	7	1	9	1	**43**
		Nitrosomonadales	*Nitrosomonadaceae*	0	0	0	1	0	0	0	0	**1**
	Gammaproteobacteria	*Aeromonadales*	*Aeromonadaceae*	0	0	0	0	0	0	15	5	**20**
		Alteromonadales	*Alteromonadaceae*	0	0	0	0	0	0	1	0	**1**
		Cellvibrionales	*Halieaceae*	5	3	13	5	15	2	11	7	**61**
			Porticoccaceae	0	1	1	0	0	1	1	0	**4**
			Spongiibacteraceae	0	0	1	0	0	0	0	0	**1**
		Enterobacteriales	*Enterobacteriaceae*	3	2	0	1	2	0	6	6	**20**
		Gammaproteobacteria_unclassified	Gammaproteobacteria_unclassified	1	0	0	0	0	0	1	0	**2**
		KI89A_clade	KI89A_clade_fa	0	0	0	0	2	0	2	0	**4**
		OM182_clade	OM182_clade_fa	0	0	0	1	0	1	0	1	**3**
		Oceanospirillales	*Litoricolaceae*	1	1	0	0	1	0	0	0	**3**
			Pseudohongiellaceae	0	0	0	0	1	0	1	0	**2**
		Pseudomonadales	*Moraxellaceae*	2	1	2	0	2	2	7	0	**16**
			Pseudomonadaceae	0	1	0	0	2	0	3	0	**6**
		SAR86_clade	SAR86_clade_fa	0	0	0	0	0	0	4	2	**6**
		Thiomicrospirales	*Thioglobaceae*	12	3	9	2	17	4	21	8	**76**
		UBA4486	UBA4486_fa	0	0	0	0	0	0	1	0	**1**
		Vibrionales	*Vibrionaceae*	1	0	0	0	0	0	0	0	**1**
Verrucomicrobia	*Verrucomicrobiae*	*Opitutales*	*Puniceicoccaceae*	6	1	3	2	4	4	8	2	**30**
		Pedosphaerales	*Pedosphaeraceae*	0	1	0	0	0	0	1	1	**3**
				96	39	89	21	113	33	162	57	**610**

Supplementary Table 3. The 30 most abundant COGs found in the SAR202 genomes obtained in this study and the number of proteins assigned to each COG.

COG IDs	Description of COG	No. of CDS			
		JH545	JH639	JH702	JH1073
COG4948	L-alanine-DL-glutamate epimerase or related enzyme of enolase superfamily	80	80	79	79
COG1028	NAD(P)-dependent dehydrogenase, short-chain alcohol dehydrogenase family	44	44	44	45
COG0477	MFS family permease	42	42	41	42
COG0642	Signal transduction histidine kinase	40	41	41	40
COG0451	Nucleoside-diphosphate-sugar epimerase	24	24	24	24
COG0745	DNA-binding response regulator, OmpR family, contains REC and winged-helix (wHTH) domain	23	23	23	23
COG3836	2-keto-3-deoxy-L-rhamnonate aldolase RhmA	19	19	19	19
COG2197	DNA-binding response regulator, NarL/FixJ family, contains REC and HTH domains	18	18	18	18
COG3119	Arylsulfatase A or related enzyme	18	18	18	18
COG0667	Predicted oxidoreductase (related to aryl-alcohol dehydrogenase)	17	17	17	17
COG0673	Predicted dehydrogenase	17	17	17	17
COG0111	Phosphoglycerate dehydrogenase or related dehydrogenase	15	15	15	15
COG1063	Threonine dehydrogenase or related Zn-dependent dehydrogenase	15	15	15	15
COG0028	Acetolactate synthase large subunit or other thiamine pyrophosphate-requiring enzyme	14	14	14	14
COG1011	FMN phosphatase YigB, HAD superfamily	14	14	14	14
COG0596	Pimeloyl-ACP methyl ester carboxylesterase	13	13	13	13
COG1312	D-mannonate dehydratase	13	13	13	13
COG1681	Archaellin (archaeal flagellin)	13	13	13	13
COG0438	Glycosyltransferase involved in cell wall biosynthesis	12	12	12	12
COG0684	Regulator of RNase E activity RraA	12	12	12	12
COG0500	SAM-dependent methyltransferase	11	11	11	11
COG2055	Malate/lactate/ureidoglycolate dehydrogenase, LDH2 family	11	11	11	11
COG2226	Ubiquinone/menaquinone biosynthesis C-methylase UbiE	11	11	11	11
COG2801	Transposase InsO and inactivated derivatives	11	11	2	3
COG2963	Transposase and inactivated derivatives	11	11	3	3
COG3386	Sugar lactone lactonase YvrE	11	11	11	11
COG0329	Dihydrodipicolinate synthase/N-acetylneuraminate lyase	10	10	10	10
COG0784	Dihydrodipicolinate synthase/N-acetylneuraminate lyase	9	9	9	9
COG2010	Cytochrome c, mono- and diheme variants	9	9	9	9
COG3618	Predicted metal-dependent hydrolase, TIM-barrel fold	9	9	9	9
	…				
	SUM of all	2273	2261	2264	2277

Supplementary Table 4 | Cellular fatty acid composition of strain JH545 and representative species of the phylum *Chloroflexota*. Strains: 1, "*Candidatus* Lucifugimonas marina" JH545 (this study); 2, *Dehalococcoides mccartyi* 195^{T} [11]; 3, *Anaerolinea thermophile* UNI-1^{T} [12]; 4, *Anaerolinea thermolimosa* IMO-1^{T} [13]; 5, *Thermosporothrix hazakensis* SK20-1^{T} [14]; 6, *Tepidiforma bonchosmolovskayae* 3753O^{T} [15]. –, not detected.

Fatty acids	1	2	3	4	5	6
**Saturated**						
$C_{12:0}$	4.1	–	1.0	1.0	–	–
$C_{14:0}$	4.3	15.7	–	5.0	–	–
$C_{15:0}$	–	–	14.0	2.0	–	–
$C_{16:0}$	10.1	22.7	35.0	16.0	10.0	–
$C_{17:0}$	–	–	7.0	3.0	1.8	–
$C_{18:0}$	2.6	16.6	12.0	3.0	7.3	–
$C_{20:0}$	–	–	–	–	–	3.9
**Unsaturated**						
$C_{16:1}$ 2-OH	–	–	–	–	9.4	–
$C_{18:1}$ ω9	–	–	–	–	0.7	–
**Branched chain**						
anteiso-$C_{13:0}$	–	–	–	3.0	–	–
iso-$C_{13:0}$	–	–	–	5	–	–
br-$C_{14:0}$	–	–	1.0	–	–	–
br-$C_{15:0}$	–	6.2	–	–	–	–
anteiso-$C_{15:0}$	–	–	–	12.0	–	–
iso-$C_{15:0}$	–	–	–	19.0	0.6	–
10-methyl $C_{16:0}$	–	25.8	–	–	1.3	–
iso-$C_{16:0}$	–	–	–	2.0	1.1	–
br-$C_{17:0}$	–	–	6.0	–	–	–
anteiso-$C_{17:0}$	–	–	6.0	21.0	10.3	–
iso-$C_{17:0}$	4.3	–	–	5.0	52.8	–
10-methyl $C_{18:0}$	20.2	–	–	–	–	–
br-$C_{19:0}$	–	–	6.0	–	–	90.4
br-$C_{21:0}$	–	–	–	–	–	4.4
**Summed feature 9***	54.4	–	–	–	–	–

*Summed features are groups of two or three fatty acids that are treated together for the purpose of evaluation in the MIDI system and include both peaks with discrete ECLs as well as those where the ECLs are not reported separately. Summed feature 9 comprises $C_{17:1}$ ω9c and/or 10-methyl $C_{16:0}$.

Supplementary References

1 Morris, R., Rappe, M., Urbach, E., Connon, S. & Giovannoni, S. Prevalence of the *Chloroflexi*-related SAR202 bacterioplankton cluster throughout the mesopelagic zone and deep ocean. *Appl. Environ. Microbiol.* **70,** 2836-2842 (2004).

2 Saw, J. H. *et al.* Pangenomics analysis reveals diversification of enzyme families and niche specialization in globally abundant SAR202 bacteria. *MBio* **11,** e02975-02919 (2020).

3 Landry, Z., Swan, B. K., Herndl, G. J., Stepanauskas, R. & Giovannoni, S. J. SAR202 genomes from the dark ocean predict pathways for the oxidation of recalcitrant dissolved organic matter. *MBio* **8,** e00413-00417 (2017).

4 Yarza, P. *et al.* Uniting the classification of cultured and uncultured bacteria and archaea using 16S rRNA gene sequences. *Nat. Rev. Microbiol.* **12,** 635-645 (2014).

5 Mehrshad, M., Rodriguez-Valera, F., Amoozegar, M. A., López-García, P. & Ghai, R. The enigmatic SAR202 cluster up close: shedding light on a globally distributed dark ocean lineage involved in sulfur cycling. *ISME J.* **12**, 655 (2018).

6 Pickl, A. & Schönheit, P. The oxidative pentose phosphate pathway in the haloarchaeon Haloferax volcanii involves a novel type of glucose-6-phosphate dehydrogenase–the archaeal Zwischenferment. *FEBS Lett.* **589**, 1105-1111 (2015).

7 Sañudo-Wilhelmy, S. A., Gómez-Consarnau, L., Suffridge, C. & Webb, E. A. The role of B vitamins in marine biogeochemistry. *Annu. Rev. Mar. Sci* **6**, 339-367 (2014).

8 Xia, Y. *et al.* Sulfide production and oxidation by heterotrophic bacteria under aerobic conditions. *ISME J.* **11**, 2754-2766 (2017).

9 Wei, Z.-F., Li, W.-L., Huang, J.-M. & Wang, Y. Metagenomic studies of SAR202 bacteria at the full-ocean depth in the Mariana Trench. *Deep-Sea Res. I: Oceanogr. Res. Pap.* **165**, 103396 (2020).

10 Schlitzer, R. Ocean Data View. https://odv.awi.de (2023).

11 Löffler, F. E. *et al. Dehalococcoides mccartyi* gen. nov., sp. nov., obligately organohalide-respiring anaerobic bacteria relevant to halogen cycling and bioremediation, belong to a novel bacterial class, *Dehalococcoidia* classis nov., order *Dehalococcoidales* ord. nov. and family *Dehalococcoidaceae* fam. nov., within the phylum *Chloroflexi*. *Int. J. Syst. Evol. Microbiol.* **63**, 625-635 (2013).

12 Sekiguchi, Y. *et al. Anaerolinea thermophila* gen. nov., sp. nov. and *Caldilinea aerophila* gen. nov., sp. nov., novel filamentous thermophiles that represent a previously uncultured lineage of the domain *Bacteria* at the subphylum level. *Int. J. Syst. Evol. Microbiol.* **53**, 1843-1851 (2003).

13 Yamada, T. *et al. Anaerolinea thermolimosa* sp. nov., *Levilinea saccharolytica* gen. nov., sp. nov. and *Leptolinea tardivitalis* gen. nov., sp. nov., novel filamentous anaerobes, and description of the new classes *Anaerolineae* classis nov. and

Caldilineae classis nov. in the bacterial phylum *Chloroflexi*. *Int. J. Syst. Evol. Microbiol.* **56**, 1331-1340 (2006).

14 Yabe, S., Aiba, Y., Sakai, Y., Hazaka, M. & Yokota, A. *Thermosporothrix hazakensis* gen. nov., sp. nov., isolated from compost, description of *Thermosporotrichaceae* fam. nov. within the class *Ktedonobacteria* Cavaletti *et al*. 2007 and emended description of the class *Ktedonobacteria*. *Int. J. Syst. Evol. Microbiol.* **60**, 1794-1801 (2010).

15 Kochetkova, T. V. *et al. Tepidiforma bonchosmolovskayae* gen. nov., sp. nov., a moderately thermophilic *Chloroflexi* bacterium from a Chukotka hot spring (Arctic, Russia), representing a novel class, *Tepidiformia*, which includes the previously uncultivated lineage OLB14. *Int. J. Syst. Evol. Microbiol.* **70**, 1192-1202 (2020).
