# Supplementary material for: Cultivation of marine bacteria of the SAR202 clade
Source: Nat Commun. 2023 Aug 22;14:5098. doi: 10.1038/s41467-023-40726-8 (PMC10444878; doi:10.1038/s41467-023-40726-8)
Supplement: Supplementary file 3 — Description of Additional Supplementary Files [file 41467_2023_40726_MOESM3_ESM.pdf]

Description of Additional Supplementary Files:

Supplementary Data 1: Information on the metabolic pathway steps of strain JH545 indicated by the numbers in Fig. 3.

Supplementary Data 2: Metagenome samples used in fragment recruitment (Fig. 6).
