# Supplementary material for: Cultivation of marine bacteria of the SAR202 clade
Source: Nat Commun. 2023 Aug 22;14:5098. doi: 10.1038/s41467-023-40726-8 (PMC10444878; doi:10.1038/s41467-023-40726-8)
Supplement: Supplementary file 6 — Reporting Summary [file 41467_2023_40726_MOESM6_ESM.pdf]

nature portfolio

Corresponding author(s): Jang-Cheon Cho

Last updated by author(s): Jul 31, 2023

Reporting Summary

Nature Portfolio wishes to improve the reproducibility of the work that we publish. This form provides structure for consistency and transparency in reporting. For further information on Nature Portfolio policies, see our Editorial Policies and the Editorial Policy Checklist.

Statistics

For all statistical analyses, confirm that the following items are present in the figure legend, table legend, main text, or Methods section.

n/a	Confirmed	
☐	☒	The exact sample size (*n*) for each experimental group/condition, given as a discrete number and unit of measurement
☐	☒	A statement on whether measurements were taken from distinct samples or whether the same sample was measured repeatedly
☐	☒	The statistical test(s) used AND whether they are one- or two-sided<br>*Only common tests should be described solely by name; describe more complex techniques in the Methods section.*
☒	☐	A description of all covariates tested
☒	☐	A description of any assumptions or corrections, such as tests of normality and adjustment for multiple comparisons
☐	☒	A full description of the statistical parameters including central tendency (e.g. means) or other basic estimates (e.g. regression coefficient) AND variation (e.g. standard deviation) or associated estimates of uncertainty (e.g. confidence intervals)
☐	☒	For null hypothesis testing, the test statistic (e.g. *F*, *t*, *r*) with confidence intervals, effect sizes, degrees of freedom and *P* value noted<br>*Give P values as exact values whenever suitable.*
☒	☐	For Bayesian analysis, information on the choice of priors and Markov chain Monte Carlo settings
☒	☐	For hierarchical and complex designs, identification of the appropriate level for tests and full reporting of outcomes
☒	☐	Estimates of effect sizes (e.g. Cohen's *d*, Pearson's *r*), indicating how they were calculated

Our web collection on statistics for biologists contains articles on many of the points above.

Software and code

Policy information about availability of computer code

Data collection	Cell counts of the growth experiments were obtained using Guava CytoSoft (v5.3).
Data analysis	Prokka (v1.12); IMG-ER (https://img.jgi.doe.gov/cgi-bin/mer/main.cgi); eggnog-mapper (v2.0.1); hmmsearch (v3.3); dbCAN2 (http://cys.bios.niu.edu/dbCAN2); IslandViewer 4 (https://www.pathogenomics.sfu.ca/islandviewer/); GTDB-Tk (v1.7.0); Mothur software package (v1.39.5); SINA online aligner (v1.2.11, http://www.arb-silva.de/aligner); MEGA software (v7.0); iTOL (https://itol.embl.de/), RAxML (v8.2.12); R environment (v4.0.2); R packages (tidyverse v1.3.2, ggplot2 v3.3.6, viridis v0.6.2, and patchwork v1.1.2); BioRender (https://biorender.com); Cytoscape (v3.7.2); Enzyme Similarity Tool (EFI-EST; https://efi.igb.illinois.edu/efi-est/); Cutadapt (v2.7); QIIME2 (v2020.11)

For manuscripts utilizing custom algorithms or software that are central to the research but not yet described in published literature, software must be made available to editors and reviewers. We strongly encourage code deposition in a community repository (e.g. GitHub). See the Nature Portfolio guidelines for submitting code & software for further information.

Data

Policy information about availability of data

All manuscripts must include a data availability statement. This statement should provide the following information, where applicable:

- Accession codes, unique identifiers, or web links for publicly available datasets
- A description of any restrictions on data availability
- For clinical datasets or third party data, please ensure that the statement adheres to our policy

All relevant data supporting the findings of this study are available within the paper and its supplementary information and data files. Source data are provided

with this paper. The 16S rRNA gene sequences of 24 isolates in SAR202 group I generated in this study have been deposited in the GenBank database under accession numbers OQ689977–OQ690000 [https://www.ncbi.nlm.nih.gov/nuccore/OQ689977.1/–https://www.ncbi.nlm.nih.gov/nuccore/OQ690000.1]. The whole genome sequences have been deposited in the GenBank database under accession numbers CP046146 (JH545) [https://www.ncbi.nlm.nih.gov/nuccore/CP046146.1/], CP046147 (JH1073) [https://www.ncbi.nlm.nih.gov/nuccore/ CP046147.1/], WMBD00000000 (JH639) [https://www.ncbi.nlm.nih.gov/nuccore/WMBD00000000.1/], and WMBE00000000 (JH702) [https://www.ncbi.nlm.nih.gov/nuccore/WMBE00000000.1/]. The genomic data are also available at IMG/M database with genome IDs 2901382945 (JH545) [https://img.jgi.doe.gov/cgi-bin/mer/main.cgi?section=TaxonDetail&page=taxonDetail&taxon_oid=2901382945], 2917498938 (JH1073) [https://img.jgi.doe.gov/cgi-bin/mer/main.cgi?section=TaxonDetail&page=taxonDetail&taxon_oid=2917498938], 2892960865 (JH639) [https://img.jgi.doe.gov/cgi-bin/mer/main.cgi?section=TaxonDetail&page=taxonDetail&taxon_oid=2892960865], and 2892963810 (JH702) [https://img.jgi.doe.gov/cgi-bin/mer/main.cgi?section=TaxonDetail&page=taxonDetail&taxon_oid=2892963810].

Research involving human participants, their data, or biological material

Policy information about studies with human participants or human data. See also policy information about sex, gender (identity/presentation), and sexual orientation and race, ethnicity and racism.

Reporting on sex and gender	Not applicable
Reporting on race, ethnicity, or other socially relevant groupings	Not applicable
Population characteristics	Not applicable
Recruitment	Not applicable
Ethics oversight	Not applicable

Note that full information on the approval of the study protocol must also be provided in the manuscript.

Field-specific reporting

Please select the one below that is the best fit for your research. If you are not sure, read the appropriate sections before making your selection.

☒ Life sciences ☐ Behavioural & social sciences ☐ Ecological, evolutionary & environmental sciences

For a reference copy of the document with all sections, see nature.com/documents/nr-reporting-summary-flat.pdf

Life sciences study design

All studies must disclose on these points even when the disclosure is negative.

Sample size	No statistical methods were used to determine sample sizes. The experiments presented in Fig. 2c, Fig. 5, ans Supplementary Fig. 3 were performed in three or two biological replicates, based on previous similar studies and practical consideration. The other experiments presented in Fig. 2a-b (growth curves of the four strains), Supplementary Table 4 (cellular fatty acid composition), and Supplementary Fig. 2 (microbial community structure of the seawater samples) were analyzed without replicates.
Data exclusions	No data were excluded from the analyses.
Replication	The experiments presented in Fig. 2c, Fig. 5, ans Supplementary Fig. 3 were performed in three or two biological replicates. All attempts at replication were successful.
Randomization	Randomization was not required in this study, because all samples (replicates) and data were analyzed and reported without subsampling or exclusion.
Blinding	Blinding was not relevant to this study, because all findings and conclusions of this study were based on objective (quantitative) measurements and analyses which did not allow for subjective judgment.

Reporting for specific materials, systems and methods

We require information from authors about some types of materials, experimental systems and methods used in many studies. Here, indicate whether each material, system or method listed is relevant to your study. If you are not sure if a list item applies to your research, read the appropriate section before selecting a response.

Materials & experimental systems

n/a	Involved in the study
☒	☐ Antibodies
☒	☐ Eukaryotic cell lines
☒	☐ Palaeontology and archaeology
☒	☐ Animals and other organisms
☒	☐ Clinical data
☒	☐ Dual use research of concern
☒	☐ Plants

Methods

n/a	Involved in the study
☒	☐ ChIP-seq
☒	☐ Flow cytometry
☒	☐ MRI-based neuroimaging
